# PINC: Pickup Non-Critical Node Based *k*-Connectivity Restoration in Wireless Sensor Networks

**DOI:** 10.3390/s21196418

**Published:** 2021-09-26

**Authors:** Vahid Khalilpour Akram, Zuleyha Akusta Dagdeviren, Orhan Dagdeviren, Moharram Challenger

**Affiliations:** 1 International Computer Institute, Ege University, 35100 Izmir, Turkey; vahid.akram@ege.edu.tr (V.K.A.); orhan.dagdeviren@ege.edu.tr (O.D.); 2 Department of Computer Science, University of Antwerp, 2020 Antwerp, Belgium; moharram.challenger@uantwerpen.be; 3 Flanders Make Strategic Research Center, 3001 Leuven, Belgium

**Keywords:** Wireless Sensor Networks, connectivity restoration, *k*-connectivity, mobility, reliability, minimum vertex cut, fault tolerance

## Abstract

A Wireless Sensor Network (WSN) is connected if a communication path exists among each pair of sensor nodes (motes). Maintaining reliable connectivity in WSNs is a complicated task, since any failure in the nodes can cause the data transmission paths to break. In a *k*-connected WSN, the connectivity survives after failure in any *k*-1 nodes; hence, preserving the *k*-connectivity ensures that the WSN can permit *k*-1 node failures without wasting the connectivity. Higher *k* values will increase the reliability of a WSN against node failures. We propose a simple and efficient algorithm (PINC) to accomplish movement-based *k*-connectivity restoration that divides the nodes into the critical, which are the nodes whose failure reduces *k*, and non-critical groups. The PINC algorithm pickups and moves the non-critical nodes when a critical node stops working. This algorithm moves a non-critical node with minimum movement cost to the position of the failed mote. The measurements obtained from the testbed of real IRIS motes and Kobuki robots, along with extensive simulations, revealed that the PINC restores the *k*-connectivity by generating optimum movements faster than its competitors.

## 1. Introduction

Wireless Sensor Networks (WSNs) are incorporate sensing devices (motes) that gather data from the environment, process the collected data and deliver them to interested parties. WSNs are broadly utilized in many areas, including agriculture, industrial manufacturing and automation, disaster control, military, health care, and structural monitoring [1,2,3,4]. Generally, WSNs have no strong and confident communication infrastructure and all nodes work as an endpoint for data collection and also as an intermediate carrier on a communication path to transmit the packets between other nodes. Generally, to develop and extend a WSN with new nodes, we place additional relay nodes in the transmission range of existing nodes that are already a part of the WSN. This process considerably simplifies the establishment of WSNs in harsh environments but, at the same time, produces new kinds of difficulties and impediments. Using other sensor nodes for delivering the data messages between remote nodes increases the risk of network segmentation. In an arbitrary connected network, if a sensor node stops running for any reason (device crash, battery draining, etc.), the transmission routes between other existing motes can be eliminated. Put differently, falling some motes can break the WSN into isolated fragments. Generally, in WSNs, we have one or more special sink nodes that gather the collected data and instructions between the motes and the interested parties. Breaking the connectivity of some nodes may make a group of nodes unreachable from the *sink* and wastes several effective devices.

In a 1-connected network, we have some special nodes, called the cut vertices, whose failure simply divides the network into disconnected fragments. Hence, the connectivity of sensor nodes in 1-connected networks is usually unpredictable, since the connectivity of the whole WSN relies on the precise operation of a few sensor nodes. In a 2-connected WSN, at least two motes must fail to drop all communication routes to a subset of operational nodes. Generally, to break the connectivity of a *k*-connected WSN, at least *k*-1 motes must terminate execution.

Preserving the *k*-connectivity ensures that the WSN can permit the failure of up to *k*-1 sensor nodes without losing connectivity. In every *k*-connected network, we have at least *k* nodes whose failure reduces *k* value by 1. We call these nodes critical because their failure reduces the fault tolerance. In this paper, we study the movement-based connectivity restoration problem, in which mobile nodes move to necessary positions to restore the connectivity status of a WSN. The contributions of this paper are listed as follows:

- We propose a pickup non-critical node based *k*-connectivity restoration algorithm (PINC) that identifies the critical nodes and then generates minimum-cost movements for *k*-connectivity restoration when a critical node stops working.
- We theoretically prove the correctness of the proposed algorithm. We also show, from our complexity analysis, that the time complexity of the proposed algorithm is better than its counterparts.
- We implement the PINC algorithm on a testbed of Kobuki robots and IRIS sensor motes. To obtain results from large-scale networks, we provide extensive simulations. From the obtained measurements, we found that the PINC performs very well in terms of movement, cost and time.

The remaining sections of the paper are organized as follows: In Section 2, we study the relevant existing research on the *k*-connectivity restoration problem. Section 3 presents essential background information and a formal definition of the problem. In Section 4, the proposed algorithms have been presented. The complexity analysis and proof of correctness are studied in Section 5. Section 6 includes the testbed and simulation results of the algorithm. Finally, conclusions have been drawn in Section 7.

## 2. Related Work

Keeping the entire WSN connected is an indispensable objective for many application setups. To accomplish this, a configuration aiming to improve the sink node’s reliability and reduce the maintenance cost was given in [5]. Wang et al. proposed a wolf pack optimization approach to improving coverage and providing connectivity in mobile sensor networks [6]. The authors designed a strategy to achieve no-gap and minimum overlap for the sensing area. Energy efficiency is a crucial objective for WSNs, as nodes are battery powered [7,8,9,10,11,12,13]. In [14], the connectivity and efficiency algorithm was proposed to reduce energy consumption and provide network connectivity. A relationship was derived among the connectivity, transmission range and the node count. Yan et al. designed a deployment approach to tackle the energy holes problem and improve the reliability and coverage of underwater WSNs [15]. The authors presented a growth ring style-based method to form a connected tree layout. In [16], authors proposed a connectivity restoration approach based on machine learning and aimed to decrease the energy consumption. The designed strategy uses a radial basis function neural network with an unscented Kalman Filter. Baroudi et al. proposed a fuzzy-logic-based node relocation technique to maintain the connectivity of a WSN, along with other optimization objectives [17]. The authors evaluated their presented algorithm on a testbed of Khepera IV robots. In [18], a position-aware connectivity restoration algorithm was given for WSNs. This method converts a sensor node with lower energy than the predefined threshold to a recovery coordinator. Liu et al. proposed a connectivity restoration technique for underwater WSNs by utilizing the minimum number of relay nodes [19]. The integer nonlinear programming formulation was given along with a heuristic algorithm. In [20], a centrality-based connectivity restoration approach was presented. The designed strategy regards the previous positions of the upstream nodes and can handle multiple node failures. Liu et al. proposed a connectivity establishment approach for disconnected segments of WSNs [21]. To evaluate and select the segments, three different approaches were presented by the authors. Zhang et al. [22] designed a connectivity restoration approach to WSNs that aims to minimize the loss of coverage area. Backup nodes are utilized by the proposed approach to deal with the node failures. In [23], a connectivity restoration algorithm was presented that can avoid the obstacles presented in the sensing area. The relay nodes are located by the algorithms, according to a Steiner-Tree-based approach. The aforementioned studies aim to provide the connectivity (where *k* = 1) of a WSN, whereas the algorithm proposed in this paper restores *k*-connectivity for general *k* values.

Generally, the research on *k*-connectivity focuses on *establishment*, *detection* or *restoration* problems. In the *k*-connectivity establishment problem, the aim is to deploy *k*-connected WSNs with a given number of nodes. The sensor nodes are placed using predefined rules or with specific patterns [24,25,26,27,28,29,30] and calculating precise communication power to set the transmission range of the motes [31,32,33,34,35,36,37] are the main methods used to deploy *k*-connected networks.

In the *k*-connectivity detection problem, we try to find (detect) the *k* value of the WSN. There are various algorithms that can detect the *k* of a given graph in polynomial time [38,39]. There are some other approaches that input a *k* and ensure that the provided network is *k*-connected [39,40]. The distributed *k*-connectivity approaches find an estimation of *k* [41,42,43,44,45] or determine the exact *k* value by sending extra messages [46].

The aim of *k*-connectivity restoration is to preserve the *k*-connectivity of the WSN after losing some motes. Deploying new motes [47,48,49,50,51,52], extending the transmission ranges [53] and moving the available motes [54,55,56,57,58,59] are significant approaches to connectivity restoration. Most of the proposed approaches restore 1-connectivity after failure in a cut vertex [54,55,57,58,59,60]. This paper focuses on the movement-based *k*-connectivity restoration problem.

To restore the *k*-connectivity of a given WSN, an algorithm named MCCR has been proposed in [56]. This algorithm uses maximum weighted matching and *k*-connectivity test algorithms to output optimal movements. After failure in a node, the MCCR algorithm constructs a *P* set from the position of all motes and another set *V* of operating nodes. Therefore, we have |P|=1+|V|. The MCCR algorithm deletes a position from *P* and checks whether the remaining positions form a *k*-connected graph. If the remaining positions create a *k*-connected network, the MCCR generates a matching over the sets *P* and *V* by creating an edge between each node and its neighbor positions. After that, the algorithm discovers the maximum weighted matching in the obtained bipartite graph. In this graph, the weights of the edges among each v∈V and p∈P are set as the reciprocal of the moving cost of node *v* to the position *p*. This operation continues for all positions and finally, the match with the highest value is chosen as the optimum movement. If removing every position from *P* violates the *k*-connectivity of the graph, MCCR concludes that, with the remaining active nodes, *k*-connectivity restoration is impossible. Considering that the matching algorithm’s time complexity is $O(n^{3})$ [61] and the fastest *k*-connectivity testing the algorithm’s time complexity is O(mnk) [39], the time complexity of MCCR will be $O(n\times max\left\{mnk,n^{3}\right\})$.

TAPU was designed for *k*-connectivity restoration, which establishes a shortest-path tree and selects the nearest safe mote for moving [62]. After failure in a node, TAPU calls a *k*-connectivity test algorithm to check whether the failed mote does not decrease the *k* value. The algorithm immediately finishes if the failed node has no effect on *k* value. Otherwise, the algorithm establishes a shortest-path tree rooted at the failed node. Starting from the closest node to the failed node’s position, the algorithm moves each node to its parent location until the *k* is restored to its original value. After moving each node, the algorithm uses a *k*-connectivity test approach with O(mnk) time to check whether the network is *k*-connected. In the worst case, n−1 motes may move to the new position, which increases the time complexity of TAPU to $O(mn^{2}k)$. A distributed approach to *k*-connectivity restoration has been proposed in [63]. This approach models a heterogeneous WSN, in which some nodes are mobile and some nodes are static. In addition, the authors accept that the general WSN topology is not available and the motes only know their local neighborhoods. The algorithm, which is called CMH, detects the nodes whose failure reduces *k* using the local neighborhood information and the remote disjoint paths between some nodes. The mobile nodes broadcasts their location to the entire network, so each node may call mobile node closest to its failed neighbor node. To prevent unnecessary movements, each node estimates its failure effect on the *k* value and shares this information with its neighbors. The CMH algorithm cannot find the optimum movements and has $O(n\delta k^{2})$ time and message complexity, where δ is the WSN’s maximum node degree. The proposed algorithm in this paper benefits from the minimum vertex cut information and finds optimum movements with $O(2^{k}n^{3})$ time complexity.

## 3. Problem Formulation

**Figure 1:**
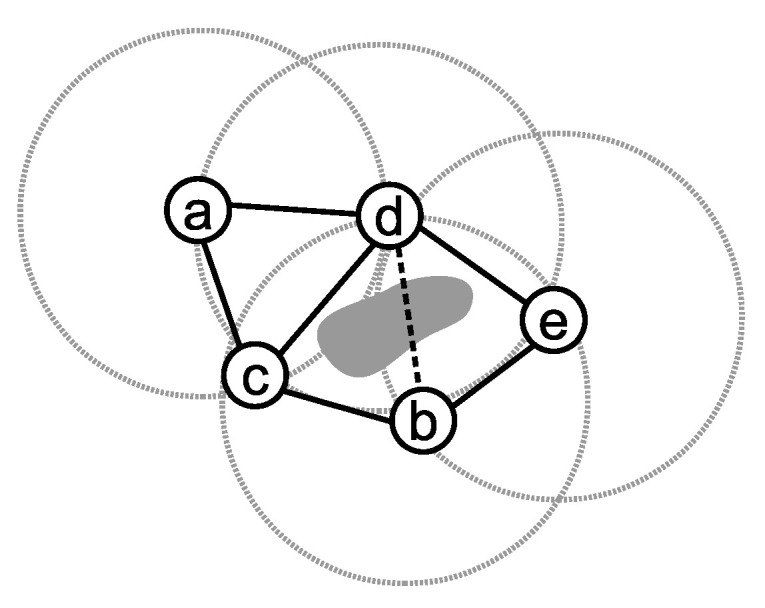
A sample multi-hop network.

Formally, a graph *G* is *k*-connected if it protects its connectivity after deleting any *k*-1 vertices. A graph G(V,E) can be good representation of a WSN, where *V* and *E* are the set of nodes and edges, respectively. Generally, in WSNs, the nodes located in the transmission range of each other have a communication channel and can send data messages to each other. In Figure 1, we have an example of a two-connected WSN with five motes, where *V* consists of *a*, *b*, *c*, *d* and *e*, and *E* includes (a,d),(a,c),(d,c),(d,e),(b,e) and (b,c). The dashed circles around the nodes show their radio ranges. In this network, failure in nodes *b*, *c*, *d* or *e* reduces the *k* to 1.

We may assume that the motes can detect and ignore the links passing from obstacles (dashed edge in Figure 1). Several techniques, such as utilizing ultrasonic waves or using received single strength indicator, can be used to identify the obstacles [64,65,66,67,68,69,70,71]. Ignoring the links passed over obstacles simplifies the communication and movement model. In this manner, the motes with communication links may directly move to the position of others. For example, in Figure 1, we assume that nodes *b* and *d* detect the obstacle and ignore link (b,d). Therefore, nodes *i* and *j* each other’s position if (i,j)∈E.

As mentioned previously, in a *k*-connected network, at east *k*, a disjoint paths exists among every pair of nodes. A path p(s,t) is an ordering of vertices that connect nodes *s* and *t* to each other. Except for the starting and ending nodes, two disjoint paths do not share common vertex. In Figure 1, for example, $p_{1}\left(a,e\right)=\left\{a,c,b,e\right\}$ and $p_{2}\left(a,b\right)=\left\{a,d,e\right\}$ are two disjoint paths between *a* and *e* and there is no other disjoint path between them.

The smallest subset of nodes whose removal breaks a network into disconnected fragments is defined as *Minimum Vertex Cut*. A minimum vertex cut of any *k*-connected network has exactly *k* elements and a network may have more than one minimum vertex cut. In Figure 1, for example, {c,d}, {b,d} and {c,e} are the minimum vertex cuts. In the remaining parts of this paper, we show the union of all minimum vertex cuts with *C* and refer to these nodes as critical nodes. Therefore, in Figure 1, the vertices in C={b,c,d,e} are critical. Failure in any critical node v∈C reduces *k* by 1 and weakens the fault tolerance of the network; hence, the nodes in *C* are critical for preserving the *k*-connectivity of a network. For example, in Figure 1, removing node *b* creates a 1-connected network, but removing node *a* has no consequence.

**Figure 2:**
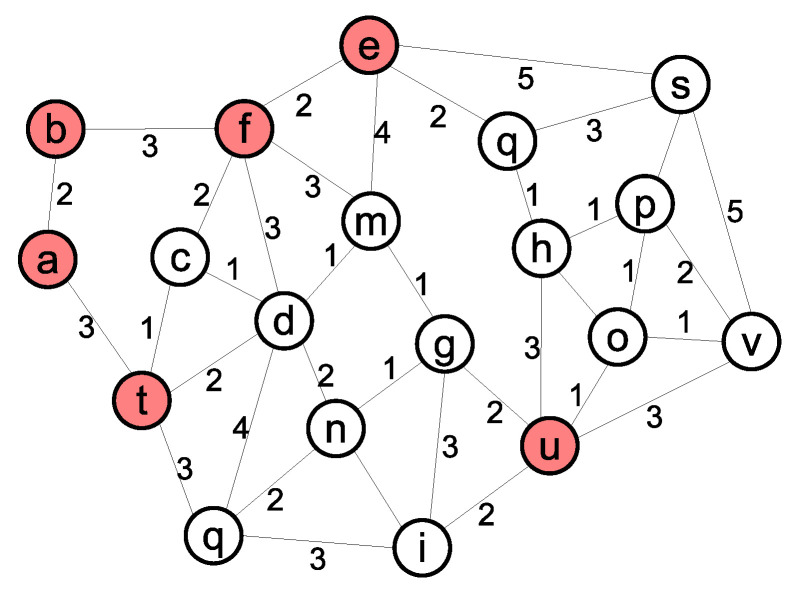
A 2-connected network with 6 critical and 13 noncritical nodes.

At least *k* critical vertices exist in a *k*-connected WSN. In our proposed algorithm, detecting the critical nodes is the first step in *k*-connectivity restoration. Failure in non-critical nodes does not affect the value of *k*, but may change the number of critical nodes. Figure 2 displays another 2-connected WSN, in which the critical nodes set is C={a,b,e,f,t,u} (red nodes). Removing any critical vertex in Figure 2 decreases the *k* value to 1, but failure in any non-critical vertex has no effect on *k*. The weights of the edges show the moving cost among its endpoints. The moving cost between two nodes depends on various parameters such as terrain type, slope, distance, etc. In this paper, the moving costs are assumed to be available.

In WSNs, the transmission ranges of nodes are usually equal and the links between them are symmetric. However, in the WSNs with asymmetric edges, we may forget the directional edges because *k*-connectivity is defined on bidirectional graphs. To find the unidirectional links, each node v∈V sends its list of neighbors to its immediate neighbors. If a mote v∈V adds *u* to its neighbor list, but its id is not included in the list received from that *u*, mote *v* reveals that its edge to *u* is asymmetric and forgets all upcoming transmissions originating from *u*. In summary, we designed our algorithms under the following assumptions.

- All motes have similar hardware and software features.
- The motes are randomly distributed in the environment (the network topology is random) and each node has a distinctive identifier.
- The transmission links between the sensor motes are bidirectional.
- The motes can move to a new position in the environment.
- The network is initially *k*-connected.
- The nodes are able to detect and forget the communication links that pass over obstacles.
- The moving cost between the position of nodes is available.

Our target is to restore the *k*-connectivity of a WSN with minimum moving cost and time consumption after the failure of a node f∈V.

## 4. Proposed Algorithm

The main idea of the PINC algorithm to move non-critical mote with minimum cost to the position of the failed critical mote. If the failed mote is non-critical, then no movement is required because the WSN is still *k*-connected. In the proposed algorithm, after failure in a critical node, say, *f*, we find the critical nodes and pick up the closest non-critical node to *f* (the mote with the lowest moving cost) to move to the position of the failed nodes. The steps of the PINC are given in Algorithm 1.

The proposed algorithm accepts the network topology before the failure as graph G(V,E), expecting a connectivity value *k*, and the id of failed node *f*. In the first step, PINC calls the minimum vertex cut algorithm [72] to find the critical nodes’ set *C* (line 3).

**Algorithm 1:** PINC Algorithm.
1:
**Algorithm PINC (G(V,E),f,k)**
2:
**Begin**
3:  C←min_vertex_cut(G(V,E)).4:  **if** |C|<k **or** V=C **then return** false.5:  **if** f∉C **then return** true.6:  **for each** v∈V **do**7:   cost[*v*] ←∞.8:   next[*v*] ←v.9:  cost[*f*]← 0.10:  S←V.11:  **while** S≠∅ **do**12:   u←v∈S with minimum cost[*v*].13:   **remove** *u* from *S*.14:   **for each** neighbor *v* of *u* in *G* **do**15:     **if** cost[*u*] + G[*u*,*v*] < cost[*v*] **then**16:      cost[*v*] ← cost[*u*] + G[*u*,*v*].17:      next[*v*] ←u.18:  u←v∈V/C with minimum cost[*v*].19:  t←u.20:  **while** t≠f **do**21:   **move** *u* to the position of next[*t*].22:   t← next[*t*].23:
**End.**



The algorithm fails immediately and returns a false result if the node count of *C* is less than *k*, which means that the requested *k* is higher than the original (before the crash) *k* value of *G* (line 4). Additionally, the restoration algorithm fails if all nodes in the graph are critical (V=C). If the failed mote is not critical, then the algorithm returns true without moving any mote because the network is already *k*-connected (line 5). Otherwise, we find the shortest paths from *f* to other vertices, except the nodes in *C*, and select the closest non-critical node, say node *v*, to *f*. We may use an algorithm based on the Dijkstra’s shortest path approach [73] to obtain all shortest paths from *f* to other nodes. To do this, we utilize the cost and next arrays to store the moving cost and the following (next) mote on the path to node *f*. Initially, the moving cost of all nodes is set as infinite and the next node of *v* is set as itself (lines 6–8). The node *f*’s moving cost to its own location is 0 (line 9). To find the cost of each node, we create a copy of *V* as the set *S* (line 10). While there is a node in *S*, we remove the node with the minimum moving cost from *S* and update the moving cost of its neighbors (lines 11–17). Let *u* be the removed node from *S*. For each neighbor *v* of *u*, if the moving cost of *u* plus the cost between *v* and *u* is smaller than the moving cost of *v*, we update the moving cost of *v* and select *u* as the next node of *v*. After calculating the moving cost of all nodes, we select the non-critical node with the minimum cost and move it to the position of *f* by following the next nodes on the path (lines 18–22).

**Figure 3:**
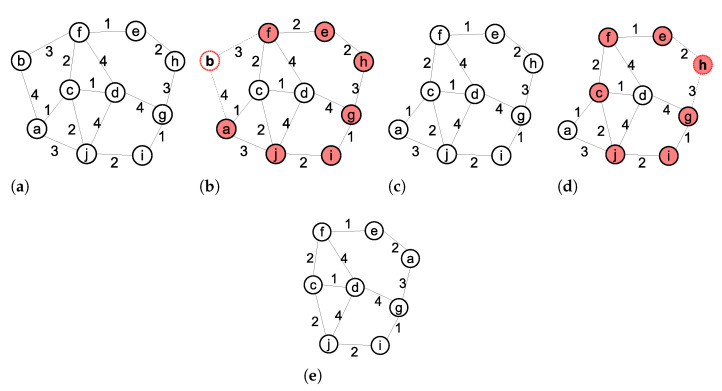
An Example operation of PINC. (**a**) Initial 2-connected network. (**b**) No movement required after failure of node *b*. (**c**) Resulting network after removing node *b*. (**d**) Failure of node h reduces *k* to 1. (**e**) Node *a* makes a movement to the location of node *h* to restore the *k* to 2.

Figure 3 shows the operations of the PINC algorithm on an example 2-connected WSN. Figure 3a shows the initial 2-connected topology and Figure 3b shows the failed node *b* and the members of the minimum vertex cut set *C* (filled nodes). We have |C|>k and C≠V so the algorithm continues to line 5. Since we have b∉C, the algorithm is true and terminates immediately without moving any node because the network is already 2-connected. Figure 3c shows the resulting network after removing node *b*. In Figure 3d, we assume that the node *h* stops working. In Figure 3d, all nodes except *a* and *d* belongs to *C*. Therefore, we have |C|>k and C≠V. In line 5, we have h∈C so the algorithm continues to line 6 and finds the shortest paths from node *h* to every node v∈V/C. In Figure 3d, we have V/C={a,d} so the shortest paths from *h* to nodes *a* and *d* are detected. The shortest path’s length from node *h* to *a* is 6. The shortest path’s length from node *h* to *d* is 7. Therefore, node *a* is moved to the position of node *h* and the network remains 2-connected (Figure 3e).

## 5. Proof of Correctness and Complexity Analysis

In this analysis section, we discuss the correctness, optimality and complexity of the proposed PINC algorithm. The following theorem proves that the PINC can restore the *k*-connectivity if at least one non-critical node exists in the network.

**Theorem** **1.** *After failure in a node, PINC correctly restores the k-connectivity of the network.*

**Proof.**  Let *G* be the graph of network topology before the failure and *f* be the failed node. The *k*-connectivity restoration is possible if, and only if, at least one node remains in *G* that can be momoved to the position of *f*, restoring the *k* to its original value. Removing any critical node c∈C reduces *k* and removing any other node v∉C has no effect on *k*. This means that we should have at least one non-critical node v∉C, such that G/v has the same *k* value as *G*. After critical node failure, PINC moves a non-critical node (if it exists) to the failed node position, which preserves the *C* set and restores the *k*-connectivity. □

**Theorem** **2.** *PINC algorithm restores the k-connectivity of any WSN with optimal movements.*

**Proof.**  The theorem is proved by contradiction. Let f∈C be the failed mote, p(f,u) be the shortest path between *f* and the closest non-critical mote u∉C to *f*. Suppose that moving the node *u* over the path from p(f,u) to the location of *f* is not an optimal solution for *k*-connectivity restoration. In this case, either there another non-critical node v∉C exists, such that moving *v* to the location of *f* is optimal solution, or there is another, shorter path between *f* and *u* in *G*.

We supposed that *u* is the closest non-critical node to *f* and, therefore, *v* is farther than *u* to *f*; hence, *v* cannot provide a shorter distance to *f*. Additionally, we find the shortest path between *f* and any other node, which means that it is impossible to find another path that is shorter than p(f,u) between *u* and *f* in *G*. Therefore, the proposed movement by PINC is an optimal solution to *k*-connectivity restoration. □

**Theorem** **3.** *The space complexity of PINC algorithm is $O(n^{2})$.*

**Proof.**  Keeping the network topology as a graph in an adjacency matrix leads to $O(n^{2})$ space complexity. The maximum size of *C* set is *n* and the shortest path and vertex separator algorithms need, at most, O(n) extra memory unit. Therefore, the space complexity of PINC is $O(n^{2})$. □

**Theorem** **4.** *The time complexity of PINC algorithm is $O(2^{k}n^{3})$.*

**Proof.**  PINC uses a minimum vertex cut algorithm whose time complexity is $O(2^{k}n^{3})$ [72]. There is a linear search in *C*, which has O(n) time complexity in the worst case. The Dijkstra algorithm $Owith(m+nlog_{2}n)$ time complexity can be used to produce the shortest paths among *f* and other nodes [74]. Therefore, the total time complexity of the PINC algorithm is $O(2^{k}n^{3})$. □

The time complexities of the existing MCCR and TAPU algorithms are $O(n\times max\{mnk,n^{3}\})$ and $O(mn^{2}k)$, respectively. Considering that the *k* value is small (usually less than 7 in most networks), the PINC algorithm is asymptotically faster than both algorithms.

## 6. Performance Analysis

**Figure 4:**
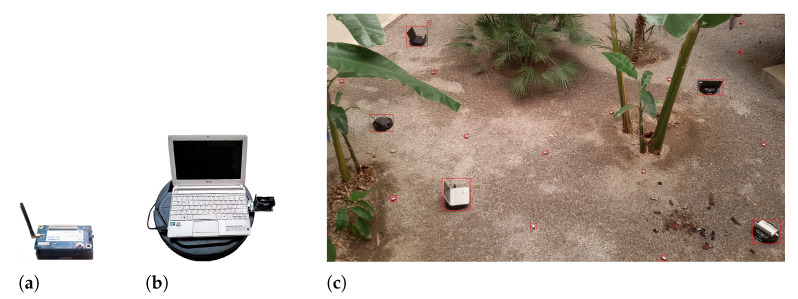
Experimental testbed hardware. (**a**) IRIS mote. (**b**) Mobile node. (**c**) Established WSN for testbed.

**Figure 5:**
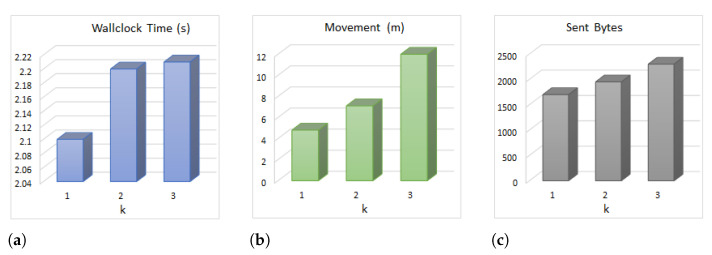
Experimental testbed measurements. (**a**) Wallclock time (s). (**b**) Movement (m). (**c**) Sent bytes.

In this section, we investigate the measurements taken from testbed experiments and simulations. The properties of the simulation environment are summarized in Table 1. For the real testbed implementation, we established networks with five mobile robots and 15 static motes as testbeds for the proposed algorithm. We used IRIS motes as static sensor motes, as shown in Figure 4a. We utilized an integration of Kobuki robots and IRIS motes as the mobile nodes given in Figure 4b. We created different topologies (Figure 4c) with *k* = 1, 2 and 3 and obtained the wallclock time, transmitted (sent) bytes and movements of the nodes. Since our mobile node count is limited, we established the topologies and stopped the nodes in such a manner that the mobile robots were selected for moving. Figure 5a shows the wallclock time of the proposed algorithm against different *k* values. The moving time of the nodes is not counted in the wallclock time. For *k* = 1 the algorithm has completed after 2.1 s, while the values for *k* = 2 and *k* = 3 are 2.19 and 2.23, respectively. Figure 5b shows the distance travelled by the nodes compared to various *k* values. Increasing the *k* value boosts critical node count; hence, the algorithm selects distanced nodes to move, which increases the distance travelled. Figure 5c depicts the cumulative sent bytes by the motes executing the proposed algorithm against the *k* value. For *k* = 1, the nodes send about 1500 bytes, while for *k* = 3, this value reaches about 2300 bytes. Increasing the *k* value boosts the average neighbor count for each node. Since each mote transmits its list of neighbors to the base station (to generate the network topology graph), in higher *k* values, more bytes are sent in the network.

To evaluate the PINC’s performance on large WSNs, we implemented MCCR, TAPU and the proposed algorithm on Java. We also implemented Proportional, Greedy and Basic central algorithms to compare their performance with the PINC algorithm. In the Proportional algorithm, the failed node’s neighbor with the smallest degree moves to the failed node’s position until the network conforms with the *k*-connectivity property. In the Greedy approach, the failed node’s nearest neighbor moves to the failed node’s position, and this operation repeats until the WSN conforms with the *k*-connectivity property. Finally, in the Basic algorithm, it is assumed that enough redundant nodes at the position of the sink node are available and after the failure of each sensor node, one of the available redundant nodes moves to the crashed node’s location.

**Figure 6:**
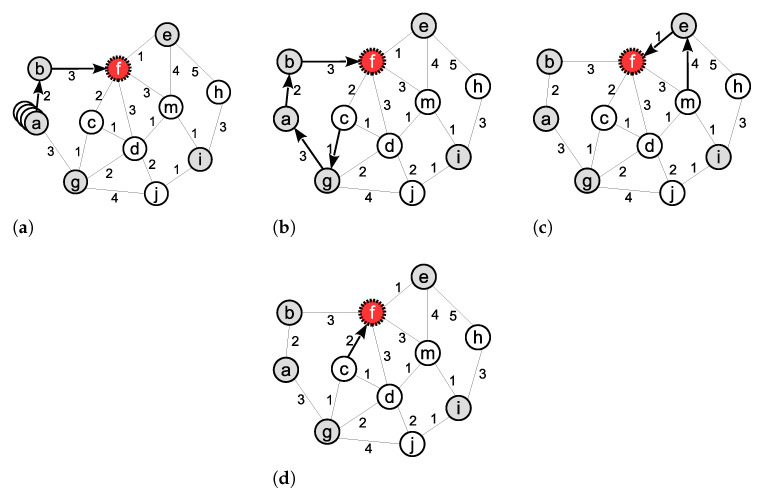
Generated movements by algorithms. (**a**) Basic algorithm. (**b**) Proportional algorithm. (**c**) Greedy algorithm. (**d**) Proposed algorithms.

Figure 6, shows the movements generated by these algorithms after failure in node *f*. In this figure, the grey nodes are critical and the white nodes are noncritical. In the Basic algorithm (Figure 6a), one of the redundant nodes near the base station (node *a*) moves to the failed node’s position. In the Proportional algorithm, (Figure 6b) node *b*, as the neighbor with the minimum degree, moves to the node *f*’s position, which leads to a chain of subsequent movements in nodes *a*, *g* and *c*. In the Greedy algorithm (Figure 6c) node *e*, as the nearest neighbor of node *f*, moves to the node *f*’s position, and node *m*, as the nearest neighbor of node *e*, moves to its position. In the proposed PINC algorithm (Figure 6d), node *c* moves to node *f*’s position.

We generated random geometric topologies with varying node counts and *k* values in a sensing field with a 1000 m × 1000 m area. In the produced graphs, the transmission range of each node is 20 m, the node counts are 50, 100, 150, 200, and 250 and the *k* values are between 1 and 5. For each WSN with a specific *k* value and specific node count, we generated 10 random instances, which leads to 250 topologies in total. To create a random geometric bidirectional topology with a specific node count and *k* value, we randomly distributed the nodes in the area by uniformly selecting their positions between 0 and 1000 and added a bidirectional edge between the nodes to ensure that their distance is less than 20 m. After that, we found the *k* value of the topology and repeated the operation if the *k* value was not equal to the desired *k*. A total of 20% of nodes were randomly selected and individually removed from the graph to simulate the failures.

**Figure 7:**
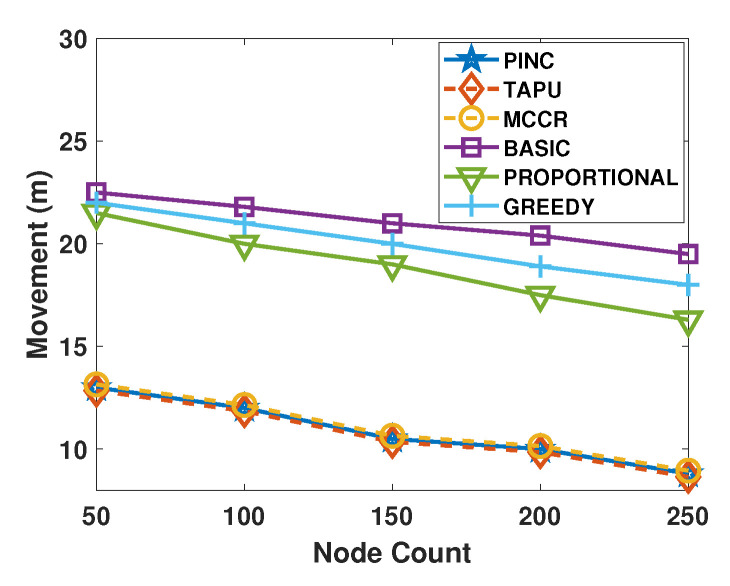
Average movements against the nodes count.

Figure 7 shows the cost of the movements generated by the algorithms against the node count. The PINC, TAPU, and MCCR produce the same movement with a lower cost than other approaches in all topologies. In the WSN with 50 nodes, the TAPU, MCCR, and PINC move the nodes about 13 m on average, while the movements generated by the Proportional, Greedy, and Basic are more than 22 m (69.2% higher). Increasing the node count decreases the distance between the nodes and the probability of critical node failure, which causes shorter movements. In the WSNs with 250 nodes, the average moving cost of MCCR, TAPU, and PINC algorithms is less than 8 m, while this value is greater than 16 m for the other approaches.

**Figure 8:**
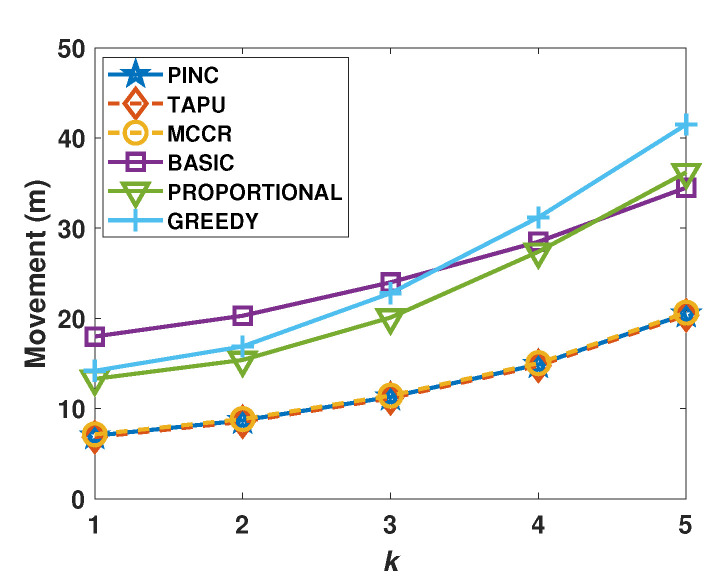
Average movements against the *k*.

Figure 8 compares the cost of the generated movements against different *k* values. The average movement cost of MCCR, TAPU, and PINC algorithms is up to 32% lower than that of other algorithms. The costs of movements generated by Basic, Greedy, and Proportional algorithms are close to each other, while the cost of movements generated by the PINC algorithm is considerably lower.

**Figure 9:**
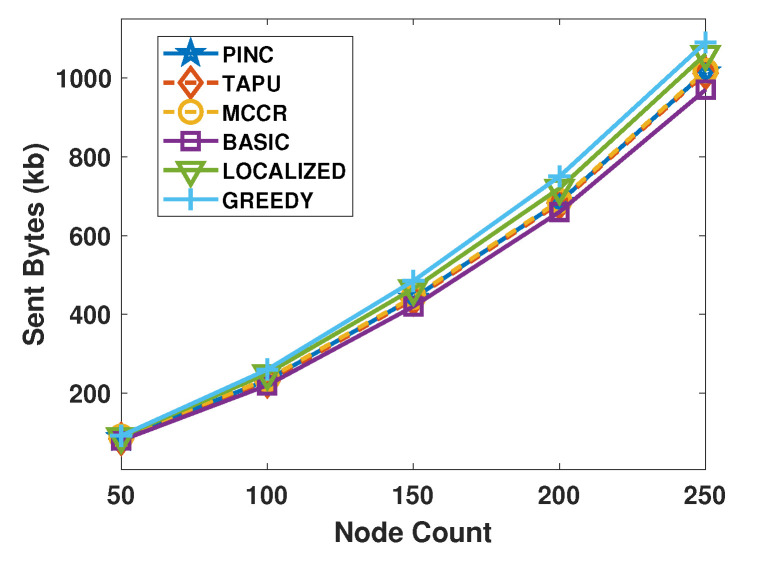
Average received bytes against the nodes count.

Figure 9 shows the average bytes sent by the implemented algorithms. All algorithms send the entire topology information to the sink node; hence, their sent bytes are close to each other. The sent bytes of the Localized and Greedy algorithms are slightly higher than PINC because they sent more messages to move more nodes in the network.

**Figure 10:**
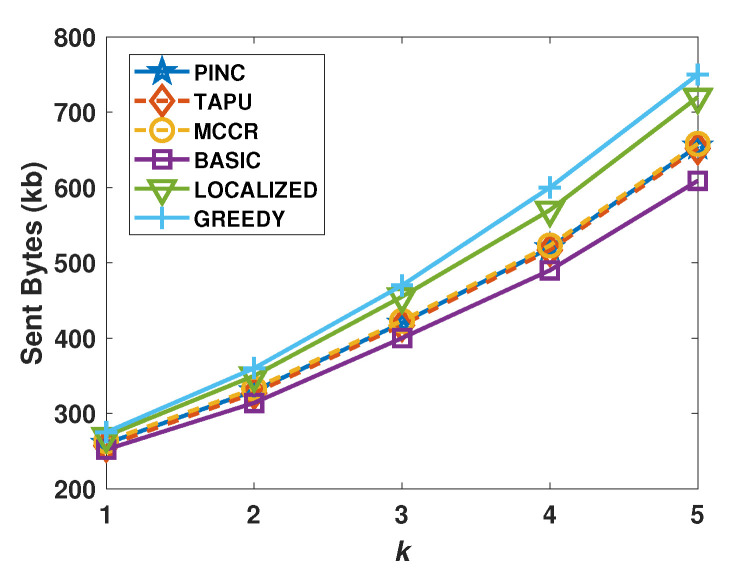
Average sent bytes against the nodes count.

Figure 10 shows the average transmitted bytes of approaches with respect to their varying *k* values. The average transmitted bytes of the Basic algorithm are lower than other algorithms, because it does not send multi-hop messages to move the remote nodes. After the Basic algorithm, the sent bytes of PINC, MCCR, and TAPU are lower than those of other algorithms.

**Figure 11:**
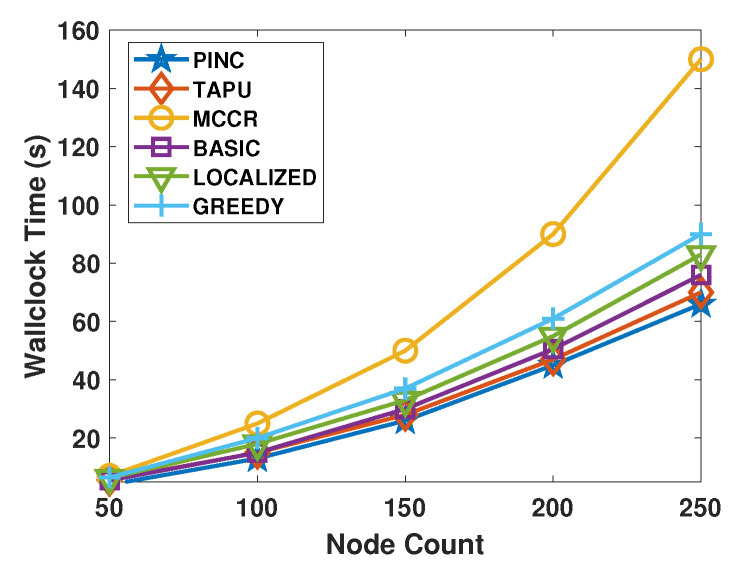
Average wallclock time against the nodes count.

**Figure 12:**
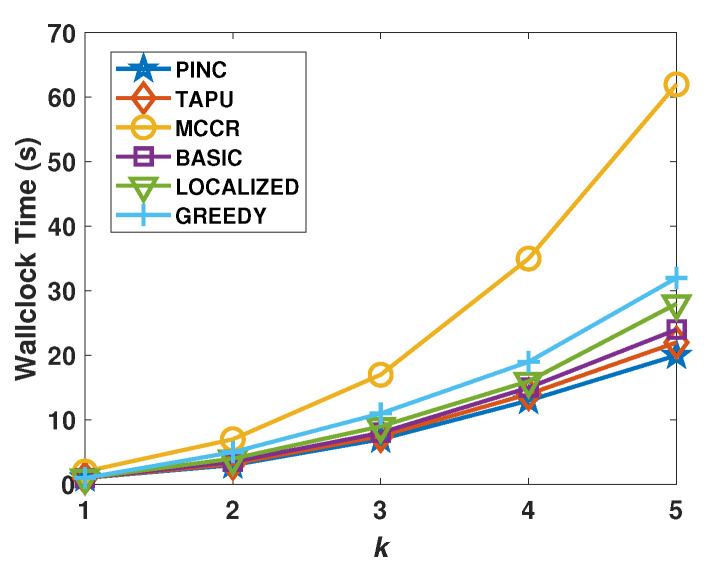
Average wallclock time against the *k*.

Figure 11 shows the wallclock time of approaches with respect to varying node counts. We did not add the moving time of nodes to the wallclock time. In the WSNs with 250 nodes, the MCCR takes approximately 150 s, which is greater than other approaches. After MCCR, the Greedy algorithm has the next longest wallclock time. In the WSNs with 250 nodes, the wallclock time of PINC is about 63 s, which is smaller than all other algorithms. Figure 12 shows the wallclock time of algorithms with respect to the varying *k* values of the networks. The MCCR has the highest wallclock time and raising the *k* value of the WSN raises its wallclock time very quickly. The PINC, TAPU, and Basic algorithms run faster than all other algorithms. Raising the *k* value has a lower effect on the wallclock time of the PINC than other algorithms. For *k* = 5, the wallclock time of PINC is about 20 s, which is 67% lower than the MCCR algorithm. The PINC, TAPU, and Basic algorithms run faster than all other algorithms. Increasing the *k* value has a lower effect on the wallclock time of the PINC than other algorithms. For *k* = 5, the wallclock time of PINC is about 20 s, which is 67% lower than the MCCR algorithm.

## 7. Conclusions

Fault tolerance and connectivity restoration are important requirements for all types of reliable networks. In mobile networks, we may move the available active nodes to the location of failed nodes to restore the lost connectivity or increase the connectivity robustness. In this paper, we proposed an efficient algorithm for the movement-based *k*-connectivity restoration problem. The proposed algorithm, named PINC, generates optimum movements to restore the *k* value of the network. PINC uses the minimum vertex cut detection algorithm to find all critical nodes whose failure decrements the *k* value. After the failure of a critical node, the PINC can restore the connectivity status by moving the closest noncritical node to the failed node’s location. We theoretically analyze the proof of correctness and time complexity of the PINC. The testbed experiment measurements and simulation results showed that PINC produces optimum movements and executes faster than the other approaches.

Besides the *k*-connectivity, different quality of service (QoS) parameters such as throughput, transmission delay, error rate, link stability, and fault tolerance should be measured appropriately to obtain a comprehensive evaluation of the network efficiency and reliability. Increasing the *k* value of the network may directly or indirectly improve the QoS parameters. For example, increasing the *k* value increases the number of disjoint paths between the nodes, which may reduce the bottlenecks and increase the transmission speed. In another example, high *k* values may improve the links’ stability and fault tolerance as the nodes can use alternate paths for communication. In this context, the effect of *k*-connectivity on different QoS parameters requires a more detailed study, which is one of the future works of this research.

Another important parameter in WSNs is the total covered area or sensing field by the nodes. Maximizing the network sensing coverage is a priority in most WSNs, as it is closely related to the connectivity. Maximizing the coverage while maintaining a reliable connectivity complicates the deployment and restoration process because, generally, increasing the distance between the nodes to maximize the sensing coverage reduces the connectivity resilience. On the other hand, establishing dense networks improves the robustness of the connectivity but reduces the overall covered area. Failure of a node may disrupt both coverage and connectivity. Moving a node with minimum moving cost can restore the connectivity but may lead to more coverage being lost. On the other hand, moving a node to maximize the coverage may lead to a a high moving cost. Although there is some research covering the 1-connectivity maintenance, the coverage aware *k*-connectivity maintenance is an open problem that can be considered in future work.

## Figures and Tables

**Table 1:** Key parameters of simulation environment.

Network Model	Geometric Undirected Weighted Graph
Number of network topologies	250
Number of nodes (*n*)	From 50 to 250 nodes
Communication range	20 m
*k*	From 1 to 5
Node distribution	Random distribution
Number of failures	20% of nodes
Area	1000 × 1000 $m^{2}$

## Data Availability

The topologies used in this paper can be downloaded from http://akademik.ube.ege.edu.tr/netos/downloads.php (accessed on 26 September 2021).
